# Randomized Controlled Trial of DHA Supplementation during Pregnancy: Child Adiposity Outcomes

**DOI:** 10.3390/nu9060566

**Published:** 2017-06-02

**Authors:** Byron A. Foster, Elia Escaname, Theresa L. Powell, Benjamin Larsen, Sartaj K. Siddiqui, John Menchaca, Christian Aquino, Rajam Ramamurthy, Daniel E. Hale

**Affiliations:** 1 Department of Pediatrics, University of Texas Health Science Center at San Antonio, San Antonio, TX 78229, USA; escaname@uthscsa.edu (E.E.); larsenbz@livemail.uthscsa.edu (B.L.); kadiwala@uthscsa.edu (S.K.S.); jamdoc@aol.com (J.M.); aquinoc@uthscsa.edu (C.A.); rramamurthy@uthscsa.edu (R.R.); hale@uthscsa.edu (D.E.H.); 2 Department of Pediatrics, University of Colorado, Aurora, CO 80045, USA; theresa.powell@ucdenver.edu

**Keywords:** docosahexaenoic acid, nutrient supplementation, pregnancy, obesity, children

## Abstract

Investigating safe and effective interventions in pregnancy that lower offspring adiposity is important given the burden of obesity and subsequent metabolic derangements. Our objective was to determine if docosahexaenoic acid (DHA) given during pregnancy to obese mothers results in lower offspring adiposity. This study was a long-term follow-up of a randomized trial of mothers with gestational diabetes or obesity who were randomized to receive DHA supplementation at 800 mg/day or placebo (corn/soy oil) starting at 25–29 weeks gestation. Anthropometric measures were collected at birth and maternal erythrocyte DHA and arachidonic (AA) levels were measured at 26 and 36 weeks gestation. At two- and four-year follow-up time points, offspring adiposity measures along with a diet recall were assessed. A significant increase in erythrocyte DHA levels was observed at 36 weeks gestation in the supplemented group (*p* < 0.001). While no significant differences by measures of adiposity were noted at birth, two or four years by randomization group, duration of breastfeeding (*p* < 0.001), and DHA level at 36 weeks (*p* = 0.002) were associated with body mass index z-score. Our data suggest that DHA supplementation during pregnancy in obese mothers may have long-lasting effects on offspring measures of adiposity.

## 1. Introduction

The importance of intrauterine nutrition and its influence on fetal programming and the development of future cardiovascular and metabolic disease have long been recognized [1]. There is now evidence to support that the intrauterine environment of a fetus not only impacts neonatal body composition but may also alter metabolic pathways that predict increased risk for glucose dysregulation and metabolic syndrome later in childhood [2]. The n-3 fatty acids, eicosapentaenoic acid (EPA) and docosahexaenoic acid (DHA), are essential nutrients derived from alpha linolenic acid or obtained from the diet. They are biologically active components of the phospholipid membrane with known effects on neuronal development and enhanced production of anti-inflammatory mediators [3,4,5,6,7]. The cardiovascular benefits of supplementation of n-3 fatty acids in children and adolescents is now the subject of recent investigations, given their role in reducing pro-inflammatory mediators [8]. Specifically, the high ratio of n-6 to n-3 fatty acids in both human and animal studies has been implicated in the increasing pro-inflammatory state in multiple conditions including type 2 diabetes mellitus and obesity [9]. This rise in the n-6/n-3 ratio of fatty acids in the Western diet corresponds with the rise in prevalence of the aforementioned conditions [9]. Alterations in the ratio of dietary n-6 to n-3 fatty acids during pregnancy in rats resulted in an increase in mean body weight in the offspring of the animals who were supplemented with a higher ratio diet during pregnancy [10]. Additionally, animal studies have shown changes in memory and cognition in rats fed a high fructose diet through modulation of the insulin receptor and synaptic plasticity that were partially counteracted with supplementation of DHA-rich diets [11].

Studies in women with gestational diabetes have shown compositional changes in the phospholipid bilayers when compared to controls, with proposed effects on receptor interactions that could alter the degree of insulin sensitivity in this population [12]. Other studies have shown that infants born to mothers with gestational diabetes may be more vulnerable to the effects of low circulating DHA through altered fetal insulin sensitivity [13]. In human cohort studies, maternal diets rich in n-3 fatty acids have been associated with decreased adiposity in early childhood. However, the cohort design of these studies leaves them vulnerable to threats to validity from confounding [14]. Other studies have found significant associations between DHA levels and fatness measured via dual-energy X-ray absorptiometry (DXA), but not by body mass index (BMI) [15]. Few randomized trials in pregnant women have tested the effect of DHA supplementation on childhood adiposity. One of the largest studies of normal weight, healthy mothers who received DHA during pregnancy was unable to show sustained differences in BMI z-scores [16]. To our knowledge, no study has examined the long-term outcomes of childhood adiposity in infants of high risk mothers who received DHA during pregnancy.

The current study evaluated the effects of DHA supplementation provided in a randomized clinical trial during pregnancy in a high-risk population of obese mothers. The primary difference between this study and the trials described above was the targeting of a high-risk group of mothers. Our objective in this analysis was to examine whether DHA supplementation had effects on measures of offspring adiposity in both the short- (birth) and long-term (early childhood).

## 2. Materials and Methods

This double-blinded, placebo-controlled, randomized clinical trial was designed to examine the effect of DHA supplementation in pregnancy (clinicaltrials.gov registry NCT00865683) on maternal insulin sensitivity. Here, we report the outcomes from that trial on infant and child growth.

### 2.1. Recruitment and Enrollment

Potential subjects were contacted at their early pregnancy pre-natal visit and screened for eligibility. Inclusion criteria were either a pre-gravid BMI ≥ 30 kg/m^2^ or gestational diabetes (GDM) and a singleton pregnancy. Exclusion criteria were high reported intake of DHA foods (i.e., more than one fish meal per week or use of any supplements that contain DHA), concurrent inflammatory, vascular, or metabolic disease, including diabetes, polycystic ovary disease, collagen vascular disease, inflammatory bowel disease, or infection, and any plans to leave the area. If they screened eligible, an initial contact visit was made at 24–26 weeks gestational age. Follow-up contacts with enrolled subjects when their children were two and four years were made by the last available phone number and address with at least three attempts made at contacting the women who had completed the study protocol during pregnancy. The women were provided a small financial incentive for participating at each follow-up visit.

### 2.2. Ethics

Informed, written consent was obtained from all participating women after careful explanation of the study. Additional informed consent was obtained at each follow-up visit at two and four years. The study protocol was approved by the Institutional Review Board at University of Texas Health Science Center, San Antonio (IRB#HCS20090506H). A second protocol for long-term follow-up at four years of age was approved by the same board (IRB#HSC20150355H).

### 2.3. Study Participants

Seventy-two women were enrolled at 25–29 weeks gestation (mean 26.6 weeks) and randomized to either placebo (corn/soy oil) or DHA (800 mg/day) supplementation (purchased from DSM Nutritional Products) for the remainder of their pregnancy. Fasting maternal blood samples were collected at enrollment and at 36 weeks gestation from 63 women who completed the study. All women were also seen at 30 weeks gestation for a compliance check. Sixty-three women completed all three required visits during their pregnancy and were considered completers of the supplementation; they are the focus of this analysis.

### 2.4. Clinical Visits with the Offspring (Children)

Sixty-three offspring of enrolled mothers who completed the study were measured at birth. Growth measurements collected at birth included infant weight, length, and head circumference. The study procedures during the follow-up appointments at two and four years post-enrollment consisted of anthropometric measurements (height, weight, waist circumference, upper arm circumference, leg circumference, arm skinfold thickness, and leg skinfold thickness) and a food frequency questionnaire at four years of age. Additionally, the Bayley Scales of Infant and Toddler Development, Third Edition (BSITD-III) were administered at the two-year visit by a trained psychologist.

### 2.5. Anthropometrics

Infant measurements of weight, length, and head circumference were collected at birth from the hospital birth records. At two and four years of age, each child participant was weighed on a digital scale with no shoes and measured to the nearest 0.1 kg with the procedure repeated. The two weight measurements were required to be within 0.2 kg for accuracy and precision. Standing height was measured in centimeters on a stadiometer with a fixed vertical bar and an adjustable headpiece with shoes removed. Height measurement was recorded to the nearest 0.1 cm, and then the process was repeated. The two height measurements were required to be within 0.2 cm for accuracy and precision.

Skinfold measurement was obtained using a skinfold caliper. Measurements of the triceps skinfold were taken midway between the acromion and olecranon processes and, for the thigh skinfold, between the proximal border of the patella and inguinal crease of the child. The skinfold was pulled away from the participant’s arm to separate the fat from the underlying muscle. Once the caliper was removed the preceding steps were repeated for a second measurement.

Arm circumference was obtained midway between the acromion and olecranon processes with a flexible tape measure to the nearest 0.1 cm. Two arm-circumference measurements were required to be within 0.2 cm for accuracy and precision. Waist circumference was obtained with a flexible tape wrapped around the participant’s abdomen in a horizontal line from the uppermost lateral border of the right iliac crest to the umbilicus, and to the uppermost lateral border of the left iliac crest. Measurements were taken to the nearest 0.1 cm. The two waist circumference measurements were required to be within 0.2 cm for accuracy and precision.

### 2.6. Dietary Assessment

At the two-year study visit, each subject (mother) completed a survey on their reported feeding habits and reported the duration of breastfeeding for their child. At the four-year study visit, each mother completed a Block Food-Frequency Questionnaire (FFQ) [17] about their child’s eating habits over the past week.

### 2.7. Data Analysis

In order to ensure adequate DHA intake, red blood cell (RBC) DHA content was measured at baseline and at 36 weeks gestation. Fasting venous blood was drawn into an elhylene diamine tetraacetic acid-coated vacutainer tube and then placed on ice to prevent hemolysis. After centrifugation for 20 min (3000× *g* at 4 °C), the plasma was removed and the RBCs were washed three times in saline solution (0.9%). The RBCs were placed in another fresh tube of pre-filled NaCl and spun again for 4 min, discarding the supernatant. This RBC pellet was frozen at −80 °C. Fatty acid composition of the RBC membrane was determined using methylation and saponification 7 methods. Capillary gas chromatograph Shimadzu-GC201 (Shimadzu Corporation, Kyoto, Japan) and helium carrier gas were used to perform this analysis. The identification of fatty acids was performed using the retention times of authenticated fatty acid standards. A 500-threshold analysis was performed, which enables the detection of n-3 fatty acids found in concentrations of less than 1% in the RBC membrane.

Descriptive statistics were used for the analysis of neonatal data and demographic characteristics. z-scores were calculated using the World Health Organization reference data for birth outcomes or Centers for Disease Control and Prevention (CDC) reference data for two- and four-year-old anthropometric outcomes [18,19]. Linear mixed models with a random intercept were used to evaluate the clinical outcomes with the randomization group or DHA level, time (two and four years coded as intervals to evaluate period effects), and their interaction (group × time) as main effects; an association with breastfeeding duration was examined using similar modeling. Linear regression was used to analyze birth outcomes separately. Data were analyzed using SPSS v22.0 (IBM, New York, NY, USA).

## 3. Results

### 3.1. Baseline Characteristics

Seventy-two high-risk mothers (obese or with gestational diabetes) were randomized to receive either DHA at 800 mg/day or a placebo at 26 weeks gestational age, and 63 mothers completed the pregnancy visits. There were no significant differences between the completers and non-completers on any maternal characteristic including maternal age, gestational age at enrollment, number of prior pregnancies, or maternal BMI.

For mothers who completed the study visits (*n* = 63), 92% overall were Hispanic, one mother was white, non-Hispanic and four mothers were black, non-Hispanic. There were no significant differences in maternal age (*p* = 0.82) or maternal pre-pregnancy BMI (*p* = 0.33) between the randomized groups (Table 1). Additionally, there were no significant differences by randomization in maternal RBC DHA levels assessed at 26 weeks gestation (*p* = 0.79), however, there were significant differences in RBC DHA levels at 36 weeks gestation (*p* < 0.001) between the placebo and the DHA-supplemented group.

### 3.2. Birth Outcomes

At birth, there was a trend of an association between DHA supplementation with length z-score (*p* = 0.08), but no other outcomes (Table 2). Additionally, there were no significant findings when using maternal DHA levels at 36 weeks or maternal RBC AA at 36 weeks as the independent variable with outcomes of birth weight, length, and weight-for-length z-scores (see Table S1 for list of predictors and outcomes examined).

### 3.3. Offspring Anthropometrics at Two and Four Years of Age

Overall, 35% (6/17) of children at two years and 40% (8/20) of children at four years were overweight or obese using CDC BMI z-score criteria.

Using linear mixed-model analysis with the primary independent variable of randomization group, there were no significant findings for the dependent variables of BMI, weight, height, arm circumference, or arm skinfold z-scores at two and four years (Table 3).

At the two-year follow-up, there were no differences between randomization groups in length of reported breastfeeding with a mean of six months (SD = 5) overall, *p* = 0.5, *t*-test. However, a bivariate correlational analysis showed a significant association (*p* < 0.05) between the reported length of breastfeeding in months and the BMI z-score at both two- and four-year time points. Given this finding, we examined breastfeeding duration and adiposity measures using a similar linear mixed-models approach in an exploratory analysis. Both the duration of breastfeeding and RBC DHA levels had significant negative relationships with the measures of adiposity assessed; in other words, increasing BMI z-score was significantly associated with lower RBC DHA levels at 36 weeks and with duration of breastfeeding reported (Table 4). An interaction term for RBC DHA levels with the original group assignment (DHA vs. placebo) had borderline significance in the arm circumference group only.

### 3.4. Dietary Intake Assessment

To attempt to take into account the other factors influencing the children’s weight status, we assessed their reported dietary intake. At four years of age, while there were no significant differences by randomization group in the dietary intake, the reported dietary intake was generally poor with a median daily intake of 13 grams of fiber (interquartile range (IQR) 9–18) and 57 grams of total fats (IQR 30-83). Overall, the children had an adequate intake of fruits at 1.4 cups per day (IQR 0.7–2.3), but an inadequate intake of vegetables at 0.7 cups per day (IQR 0.3–1.1). A comparison by weight status at four years of age only showed a difference in sugary beverage consumption with a median of 0.39 (IQR 0.0–0.5) sodas per day in the overweight or obese children and a median of 0 (IQR 0.0–0.14) in the normal weight children (*p* = 0.02).

### 3.5. Developmental Assessment

There were no differences found using the BSITD-III at the two-year follow-up when examined by randomization group, examining cognitive (*p* = 0.66), receptive (*p* = 0.52), and expressive language (*p* = 0.98), as well as fine (*p* = 0.24) and gross motor (*p* = 0.50) domains.

## 4. Discussion

Although the effects of intrauterine environment on fetal growth and subsequent risk for later adult disease is now recognized, it remains unclear as to which nutrients may actually have an impact on future growth [1]. The neurocognitive benefit of n-3 fatty acids has now been better established [5], so our goal was to see if these same essential nutrients could play a role in sustained differences on offspring body composition. It is now recommended to optimize dietary n-3 intake in expectant mothers for its neurocognitive benefits in offspring. Extending the recommendation because of its potential impact on adiposity, anti-inflammatory effects, or impact on insulin sensitivity remains an important area of investigation.

This study was unique in that it targeted a high-risk group of mothers that were obese or had a history of gestational diabetes. We did not find a sustained difference in the BMI z-score between the offspring of mothers who received high dose n-3 supplementation versus a placebo in the primary analysis by randomization group, but we did find a significant association in the exploratory analysis taking into account breastfeeding and measured DHA levels. The negative finding by randomization group is largely consistent with the literature, where there are now multiple clinical trials which have found no effect of DHA supplementation on standard measures of childhood adiposity [16,20,21]. While there is heterogeneity in the enrollment criteria among these studies (e.g., normal weight women enrolled) and the dosage of DHA, the finding of a lack of an effect on progeny outcomes is mostly consistent, though there are notable exceptions [22].

Epidemiologic studies have found a significant association between maternal n-3 levels and childhood adiposity [23]. There are also recent data from randomized trials suggesting that epigenetic modification in the offspring may occur with DHA supplementation [24,25]. Possible explanations for these apparently inconsistent findings are that the environmental components that contribute to adiposity outweigh the influence of the epigenetic changes, or that the measures of adiposity are not sensitive enough. Women who have a healthier diet during pregnancy and higher n-3 levels in epidemiologic studies are probably more likely to also have a healthier diet post-pregnancy, influencing their breastmilk composition and feeding practices in early childhood. In our study in a high-risk population who were obese or had gestational diabetes, the fact that 40% of the children at the four-year follow-up were overweight or obese coupled with the poor reported diet highlights the challenge of interventions such as this one in making a sufficient impact to affect gross measures of adiposity.

Women whose DHA levels were higher at 36 weeks, once breastfeeding was taken into account, had children with lower adiposity at two and four years of age. Since DHA supplementation would lead to greater body stores over time, the biologic significance is that these women who were breastfeeding would be delivering a higher level of DHA in their breastmilk to their infants over a longer period, thus influencing the observed effect on adiposity. Consistent with this hypothesis, a recent study [26] found that increased breastmilk DHA content was associated with a lower BMI in childhood up to age seven, though others have found no association between measured breastmilk DHA content and childhood adiposity [27]. A general association between breastfeeding and later reduced adiposity has been observed across a wide range of studies, though unmeasured confounding has often been attributed as an explanation [28].

We did not find a significant difference in the developmental assessment at two years of age. Other groups have also failed to show a difference in neurocognitive outcomes in children after supplementation with DHA at similar levels [29]. A recent systematic review found that while there was an overall effect on cognitive development assessed in infancy, there was no discernible effect in childhood [30]. So, while the epidemiologic data support the association between higher DHA levels and cognitive outcomes, the trials of supplementation have not shown as strong of an effect.

There was significant loss to follow-up over time in this cohort. The completer-only analysis presented here has some potential for selection bias, though there were no major baseline differences between completers and non-completers. Some of the subjects had moved out of the area and many families were limited by other socioeconomic factors including lack of transportation and childcare. The original enrollment of mothers in this cohort was powered for the primary outcome of changes in maternal insulin sensitivity.

Although our study was able to complete anthropometric measurements, body fat assessments, and food intake analysis on nearly a third of the original cohort, the small sample size is a limitation. The children were measured using standard assessments of growth including weight, height, and skinfold measurements. Other more reliable measurements of adiposity including air displacement plethysmography and dual-energy X-ray absorptiometry could help further validate whether supplementation of a key nutrient during pregnancy can lead to the sustained effect of lowering childhood adiposity. Finally, we did not assess physical activity at the two- or four-year follow-up time points, a possible unmeasured confounder in the traditional view of energy balance in weight regulation.

## 5. Conclusions

Our data suggest an influence of DHA supplementation during pregnancy on childhood adiposity that may be mediated by breastfeeding. Maternal supplementation of moderate doses of DHA does not have adverse effects on the offspring up to four years of age. Further research on breastmilk DHA content and the pathway by which that may be protective in this high-risk population is warranted.

## Figures and Tables

**Table 1:** Maternal demographics and clinical characteristics by randomization group for completers only (*n* = 63).

Characteristic	DHA Supplement (*n* = 34)	Placebo (*n* = 29)	*p*-Value
Maternal age, mean (SD)	29.4 (5.1)	29.1 (5.3)	0.82
Pre-pregnancy BMI, mean (SD)	33.9 (4.2)	34.8 (3.5)	0.33
Inclusion diagnosis, % (*n*)			0.45
Obese	55.9 (19)	65.5 (19)	
Gestational diabetes	44.1 (15)	34.5 (10)	
Enrollment gestational age, weeks, mean (SD)	26.5 (0.8)	26.6 (0.9)	0.61
Enrollment BMI, mean (SD)	35.5 (4.2)	35.7 (3.6)	0.80
Maternal ethnicity, % Hispanic (*n*)	94.1 (32)	89.7 (26)	0.33
Educational status, % (*n*)			0.44
<9th grade	54.5 (18)	39.3 (11)	
Some high school	9.1 (3)	7.1 (2)	
High school diploma	24.2 (8)	25.0 (7)	
Some college or above	12.1 (4)	28.6 (8)	
Insurance, % (*n*)			0.48
None	14.7 (5)	13.8 (4)	
Public	17.6 (6)	31.0 (9)	
Private	67.6 (23)	55.2 (16)	
RBC DHA% at 26 weeks, mean (SD)	5.8 (2.1)	6.0 (2.2)	0.79
RBC DHA% at 36 weeks, mean (SD)	9.7 (2.6)	6.2 (2.2)	<0.001
RBC AA% at 26 weeks, mean (SD)	21.0 (4.6)	20.5 (4.8)	0.68
RBC AA% at 36 weeks, mean (SD)	20.0 (2.3)	21.6 (2.6)	0.01

Abbreviations: RBC DHA % = red blood cell docosahexaenoic acid as percent of total red blood cell fatty acids; RBC AA % = red blood cell arachidonic acid as percent of total red blood cell fatty acids; BMI = body mass index; SD = standard deviation.

**Table 2:** Neonatal anthropometric and clinical characteristics by randomization group.

Characteristic	DHA (*n* = 34)	Placebo (*n* = 29)	*p*-Value
Child gender, % female (*n*)	44.1 (15)	37.9 (11)	0.80
Gestational age, weeks, mean (SD)	39.3 (1.1)	39.4 (1.2)	0.54
Birth weight, g, mean (SD)	3502 (433)	3484 (411)	0.86
Birth weight z-score, mean (SD)	0.84 (1.01)	0.65 (0.76)	0.39
Length, cm, mean (SD)	51.5 (1.7)	50.9 (1.9)	0.23
Length z-score, mean (SD)	1.50 (1.08)	1.04 (0.94)	0.08
Head circumference, cm, mean (SD)	34.6 (1.3)	34.7 (0.96)	0.92
Head circumference z-score, mean (SD)	0.71 (1.08)	0.63 (0.92)	0.77
Weight-for-length z-score, mean (SD)	−0.59 (0.92)	−0.31 (0.97)	0.25
Ponderal index, mean (SD)	2.56 (0.21)	2.62 (0.23)	0.27

Raw values and z-scores adjusted for gestational age and sex are presented. SD = standard deviation; DHA = docosahexaenoic acid.

**Table 3:** Anthropometric results at birth, two, and four years of age using linear mixed models, shown as estimated marginal means (SE).

Outcome Measure	DHA	Placebo	Group *p*-Value	Time *p*-Value	Group × Time *p*-Value
Adiposity measure			0.97	<0.001	0.60
Birth weight for length z-score	−0.60 (0.16)	−0.31 (0.18)			
2-year old BMI z-score	0.48 (0.39)	0.55 (0.32)			
4-year old BMI z-score	1.24 (0.42)	0.92 (0.47)			
Weight			0.40	0.16	0.97
Birth z-score	0.84 (0.16)	0.65 (0.17)			
2-year old z-score	0.64 (0.32)	0.36 (0.28)			
4-year old z-score	1.15 (0.35)	0.85 (0.38)			
Height			0.37	<0.001	0.58
Birth z-score	1.50 (0.18)	1.04 (0.20)			
2-year old z-score	0.06 (0.38)	0.01 (0.23)			
4-year old z-score	0.50 (0.23)	0.31 (0.24)			
Models for 2 and 4 years					
BMI z-score			0.85	0.08	0.57
2-year old BMI z-score	0.57 (0.39)	0.64 (0.34)			
4-year old BMI z-score	1.30 (0.44)	1.03 (0.47)			
Arm Circumference			0.84	0.51	0.69
2-year old z-score	1.54 (0.37)	1.54 (0.35)			
4-year old z-score	1.46 (0.55)	1.20 (0.59)			
Arm Skinfold			0.48	0.57	0.21
2-year old z-score	1.41 (0.58)	1.57 (0.48)			
4-year old z-score	2.19 (0.39)	1.28 (0.43)			

Measures of BMI, arm circumference, and arm skinfold thickness calculated only at two and four years of age.

**Table 4:** Analysis of adiposity outcomes at two and four years examining the effect of breastfeeding and DHA levels using linear mixed models with adiposity dependent variables as rows, parameters tested in models as columns, and values shown as parameter estimates (SE), *p*-value.

Outcome Measure	DHA vs. Placebo Group	Time	Breastfeeding Duration	RBC DHA at 36 Weeks	DHA vs. Placebo × RBC DHA
BMI z-score	−1.35 (1.48), 0.38	−0.34 (0.29), 0.26	−0.17 (0.03), <0.001	−0.47 (0.11), 0.002	0.29 (0.16), 0.10
Arm circumference z-score	−1.58 (1.16), 0.19	0.42 (0.36), 0.27	−0.19 (0.02), <0.001	−0.44 (0.10), <0.001	0.29 (0.13), 0.05
Arm skinfold z-score	−1.11 (1.49), 0.47	−0.12 (0.41), 0.77	−0.18 (0.04), <0.001	−0.41 (0.12), 0.006	0.25 (0.17), 0.16

RBC = red blood cell; DHA = docosahexaenoic acid; BMI = body mass index.

## Supplementary Materials

The following is available online at www.mdpi.com/2072-6643/9/6/566/s1, Table S1: Mixed-model analysis outcomes using birth, two- and four-year time points.
