# Supplementary material for: Randomized Controlled Trial of DHA Supplementation during Pregnancy: Child Adiposity Outcomes
Source: Nutrients. 2017 Jun 2;9(6):566. doi: 10.3390/nu9060566 (PMC5490545; doi:10.3390/nu9060566)
Supplement: Supplementary file 1 [file nutrients-09-00566-s001.docx]

**Table S1.** Mixed-model analysis outcomes using two- and four-year follow-up time points.

| **Predictor** | **Outcome** | ***p*-Value** |
| --- | --- | --- |
|  | Birth Weight Z-Score |  |
| Randomization Group |  | 0.30 |
| RBC DHA 26 Weeks |  | 0.11 |
| RBC AA 26 Weeks |  | 0.16 |
| RBC DHA 36 Weeks |  | 0.97 |
| RBC AA 36 Weeks |  | 0.48 |
| RBC DHA Change |  | 0.83 |
| RBC AA Change |  | 0.34 |
|  | Length Z-Score |  |
| Randomization Group |  | 0.05 |
| RBC DHA 26 Weeks |  | 0.10 |
| RBC AA 26 Weeks |  | 0.26 |
| RBC DHA 36 Weeks |  | 0.31 |
| RBC AA 36 Weeks |  | 0.65 |
| RBC DHA Change |  | 0.06 |
| RBC AA Change |  | 0.20 |
|  | Head Circumference Z-Score |  |
| Randomization Group |  | 0.72 |
| RBC DHA 26 Weeks |  | 0.72 |
| RBC AA 26 Weeks |  | 0.61 |
| RBC DHA 36 Weeks |  | 0.73 |
| RBC AA 36 Weeks |  | 0.87 |
| RBC DHA Change |  | 0.59 |
| RBC AA Change |  | 0.70 |
|  | Weight-for-Length Z-Score |  |
| Randomization Group |  | 0.24 |
| RBC DHA 26 Weeks |  | 0.95 |
| RBC AA 26 Weeks |  | 0.67 |
| RBC DHA 36 Weeks |  | 0.21 |
| RBC AA 36 Weeks |  | 0.07 |
| RBC DHA Change |  | 0.29 |
| RBC AA Change |  | 0.61 |
|  | Ponderal Index (PI) Z-Score |  |
| Randomization Group |  | 0.51 |
| RBC DHA 26 Weeks |  | 0.68 |
| RBC AA 26 Weeks |  | 0.52 |
| RBC DHA 36 Weeks |  | 0.08 |
| RBC AA 36 Weeks |  | 0.40 |
| RBC DHA Change |  | 0.10 |
| RBC AA Change |  | 0.78 |

Through mixed model analysis, there were no significant differences found in the two- and four-year-old outcomes of BMI Z-Score, Weight Z-Score, Height Z-score, Arm Circumference Z-Score, and Arm Skinfold Z-Score. The first model included randomization group, time, and the interaction between randomization group and time as fixed effects, while the individual study ID was held as a random effect. The second model followed the same template including RBC DHA or AA variables, time, and the interaction between RBC DHA or AA variables and time as fixed effects, while the individual study ID was held as a random effect. The third and final model only differed by using a change variable for RBC DHA and AA from 26 weeks to 36 weeks as a fixed effect. For the mixed model analysis including newborns, two-, and four-year-olds, length z-score increased over time with no significant differences observed in the group factors/covariates of: randomization, RBC AA and DHA at 26 weeks, and RBC AA and DHA change. There were also no significant differences seen between the previous group factors/covariates and their interactions with time. Length Z-Score <0.001 for Randomization Group Model (Only significant at Time 0); Length Z-Score 0.005 for RBC AA 26 Week Model (Only significant at Time 0); Length Z-Score 0.01 for RBC DHA 26 Week Model (Only significant at Time 0); Length Z-Score <0.001 for RBC AA Change Model (Only significant at Time 0); Length Z-Score 0.001 for RBC DHA Change Model (Only significant at Time 0).
